# Association of Vitamin D Deficiency with Mortality and Cardiorenal Events in Sjögren’s Syndrome and Osteoporosis

**DOI:** 10.3390/jcm15041430

**Published:** 2026-02-12

**Authors:** Ying-Kai Chen, Chien-Lin Lu, Kuo-Cheng Lu, Yu-Chen Cheng, Wui-Chiu Mui

**Affiliations:** 1 Division of Nephrology, Department of Internal Medicine, Kaohsiung Municipal United Hospital, Kaohsiung 80457, Taiwan; ykchen0528a@kmuh.gov.tw; 2 School of Medicine, College of Medicine, Fu Jen Catholic University, New Taipei 242062, Taiwan; 096195@mail.fju.edu.tw (C.-L.L.); a01399@mail.fjuh.fju.edu.tw (Y.-C.C.); 3 Division of Nephrology, Department of Internal Medicine, Fu Jen Catholic University Hospital, Fu Jen Catholic University, New Taipei 243089, Taiwan; tch33730@tzuchi.com.tw; 4 Division of Nephrology, Department of Medicine, Taipei Tzu Chi Hospital, Buddhist Tzu Chi Medical Foundation, New Taipei 231016, Taiwan; 5 Division of Neurology, Fu Jen Catholic University Hospital, Fu Jen Catholic University, New Taipei 243089, Taiwan; 6 Department of Anesthesiology, Ditmanson Medical Foundation Chia-Yi Christian Hospital, Chiayi 600056, Taiwan

**Keywords:** Sjögren’s syndrome, vitamin D deficiency, osteoporosis, cardiovascular disease, kidney outcomes

## Abstract

**Background:** Sjögren’s syndrome (SjS) is a chronic systemic autoimmune disease associated with substantial extraglandular morbidity, including osteoporosis, cardiovascular disease, and renal involvement. Vitamin D deficiency (VDD) is highly prevalent in patients with SjS and has been linked to immune dysregulation, systemic inflammation, and adverse cardiorenal outcomes in other clinical settings. However, the prognostic significance of VDD in patients with SjS and osteoporosis remains incompletely characterized. **Methods:** We conducted a retrospective cohort study using de-identified electronic health records from the TriNetX research network between 2010 and 2024. Adult patients with SjS and osteoporosis who had at least one serum 25-hydroxyvitamin D measurement within six months before or after cohort entry were included. VDD was defined as a serum 25-hydroxyvitamin D concentration below 20 ng/mL, and vitamin D adequacy (VDA) as a concentration of 30 ng/mL or higher. Propensity score matching was performed at a 1: 1 ratio using 95 baseline covariates. The primary outcome was all-cause mortality. Secondary outcomes included major adverse cardiovascular events (MACEs), major adverse kidney events (MAKEs), and fractures. **Results:** Among 19,177 eligible patients, 1218 with VDD and 7659 with VDA met the inclusion criteria. After propensity score matching, 1067 well-balanced pairs were analyzed. Over five years of follow-up, VDD was associated with higher risks of all-cause mortality, MACEs, and MAKEs compared with VDA. In contrast, fracture risk did not differ significantly between groups. Sensitivity analyses demonstrated consistent associations across analytic approaches. **Conclusions:** In patients with SjS and osteoporosis, VDD was associated with increased risks of mortality, MACEs, and MAKEs, but not fractures. These findings suggest that VDD may serve as a marker of a high-risk clinical phenotype and may be useful for long-term risk stratification in this vulnerable population.

## 1. Introduction

Sjögren’s syndrome (SjS) is a chronic systemic autoimmune disease characterized by lymphocytic infiltration of exocrine glands, resulting in the hallmark symptoms of dry eyes and dry mouth [1]. Beyond glandular involvement, SjS is increasingly recognized as a multisystem disorder associated with substantial extraglandular morbidity, including cardiovascular disease, renal involvement, metabolic disturbances, and skeletal complications, all of which contribute to adverse long-term outcomes [2,3,4,5].

Osteoporosis is a common comorbidity in SjS, particularly among postmenopausal women and patients receiving long-term systemic corticosteroid therapy [6]. In this population, bone loss frequently coexists with broader systemic vulnerability driven by chronic inflammation, immune dysregulation, and cardiometabolic comorbidity [2,7,8,9]. Vitamin D plays a central role in bone metabolism and immune regulation, and vitamin D receptors are widely expressed in immune cells, vascular endothelium, and renal tubular epithelium [10,11,12,13]. Accordingly, vitamin D deficiency (VDD) has been associated with heightened inflammatory activity, impaired immune tolerance, and increased cardiovascular and renal risk in diverse clinical settings [14,15,16].

Patients with SjS exhibit substantially lower serum 25-hydroxyvitamin D [25(OH)D] concentrations than the general population [17]. Lower vitamin D levels in SjS have been associated with higher disease activity and a greater burden of subclinical atherosclerosis, while SjS itself is linked to elevated risks of cerebrovascular and coronary events [17,18,19,20]. In parallel, VDD has been associated with accelerated renal function decline and increased mortality in patients with kidney disease [21,22]. Collectively, these observations suggest that vitamin D status may reflect systemic disease burden rather than skeletal health alone.

Despite this growing evidence, the prognostic significance of VDD in patients with SjS and osteoporosis remains incompletely defined. While vitamin D is routinely evaluated for fracture prevention, its potential role as a marker of long-term mortality and cardiorenal risk in this high-risk population has not been adequately characterized. Therefore, we conducted a large real-world cohort study using the TriNetX research network to investigate the association between VDD and long-term clinical outcomes in patients with SjS and osteoporosis. We hypothesized that VDD would be associated with increased risks of all-cause mortality, major adverse cardiovascular events (MACEs), and major adverse kidney events (MAKEs), independent of traditional risk factors.

## 2. Materials and Methods

### 2.1. Study Design and Data Source

This retrospective cohort study was conducted using de-identified patient-level data obtained from the TriNetX platform, a federated global health research network that integrates real-world electronic health records (EHRs) from hospitals and healthcare systems across multiple geographic regions, including North America, Europe, Asia-Pacific, and Latin America. The TriNetX platform enables distributed analyses across contributing healthcare organizations while maintaining data deidentification and local data governance.

All analyses were performed within the secure TriNetX cloud-based analytic environment using standardized federated queries. The database contains comprehensive longitudinal clinical information, including demographics, diagnoses, procedures, medication prescriptions, healthcare utilization records, and laboratory test results.

Because all data were fully de-identified in compliance with the Health Insurance Portability and Accountability Act (HIPAA) and the General Data Protection Regulation (GDPR), informed consent was waived. The study protocol was reviewed and approved by the institutional review board of Taipei Tzu Chi Hospital and conducted in accordance with the principles of the Declaration of Helsinki (Approval No. 13-IRB138).

### 2.2. Study Population and Inclusion Criteria

Patients with SjS were identified using the International Classification of Diseases, Tenth Revision, Clinical Modification (ICD-10-CM) code M35.00 in the TriNetX cohort between 1 January 2010 and 1 January 2024. Among these individuals, patients with a documented diagnosis of osteoporosis were further selected to construct the source population.

To define baseline vitamin D exposure, inclusion required at least one serum 25-hydroxyvitamin D measurement obtained within a predefined enrollment window spanning from six months before to six months after the diagnosis of SjS with osteoporosis. When multiple measurements were available within this window, the value closest to the cohort entry date was selected to represent baseline vitamin D status. Patients were classified into two exposure groups based on this measurement: VDD, defined as a serum 25-hydroxyvitamin D concentration < 20 ng/mL, and vitamin D adequacy (VDA), defined as a serum 25-hydroxyvitamin D concentration of ≥30 ng/mL. To enhance exposure stability and reduce misclassification, individuals with discordant measurements crossing these thresholds during the enrollment window were excluded. Additional exclusion criteria included age < 20 years at cohort entry and a history of neoplasm of bone or articular cartilage, including malignant and benign tumors (e.g., ICD-10-CM codes C41.0 and D16.0–D16.9).

### 2.3. Index Date and Follow-Up

The index date was defined as the date of cohort entry, corresponding to the first documented diagnosis of SjS with osteoporosis that satisfied all inclusion and exclusion criteria. Follow-up commenced on the day after the index date to ensure appropriate temporal separation between exposure assessment and outcome ascertainment. Patients were followed for up to five years or until the earliest occurrence of an outcome event, death, loss to follow-up, or the end of available electronic health records. To ensure accurate capture of incident events, patients with a documented history of any study outcome prior to the index date were excluded from the analysis of that specific outcome.

### 2.4. Outcome Measures

The primary outcome was all-cause mortality, defined as death recorded in the TriNetX mortality table. Secondary outcomes included MACEs, MAKEs, and fractures. MACE was defined as a composite outcome of acute myocardial infarction (ICD-10-CM I21.x–I22.x), heart failure hospitalization (ICD-10-CM I50.x), ischemic stroke (ICD-10-CM I63.x), and intracerebral hemorrhage (ICD-10-CM I61.x). MAKE was defined as a composite outcome of acute kidney injury (ICD-10-CM N17.x), chronic kidney disease stage 3–5 (ICD-10-CM N18.3–N18.6), and dialysis dependence (ICD-10-CM Z99.2). Fractures were identified using ICD-10-CM codes S12.x, S22.x, S32.x, S42.x, S52.x, S62.x, S72.x, S82.x, and S92.x. All outcome definitions followed standardized TriNetX terminologies and were uniformly applied across cohorts to ensure consistent outcome ascertainment.

### 2.5. Propensity Score Matching and Handling of Confounders

To reduce baseline imbalances and mitigate potential confounding, 1:1 propensity score matching was performed using a greedy nearest-neighbor algorithm with a caliper width of 0.1 pooled standard deviations of the logit of the propensity score. The propensity score was estimated using a multivariable logistic regression model incorporating 95 clinically relevant covariates measured within the three years preceding the index date.

These covariates included demographic characteristics (age, sex, race, and ethnicity), comorbid conditions, medication use, healthcare utilization metrics, and laboratory parameters reflecting renal function, inflammatory status, and metabolic profiles.

After matching, 1067 patients were retained in each exposure cohort. Covariate balance was assessed using standardized mean differences (SMDs), with an SMD < 0.1 considered indicative of acceptable balance. Missing data were handled using available-case analysis as implemented by the TriNetX platform, without imputation.

### 2.6. Statistical Analyses

Baseline characteristics were summarized using descriptive statistics. Continuous variables were expressed as means with standard deviations, and categorical variables were reported as counts with percentages. Time-to-event analyses were conducted using Kaplan–Meier survival curves and compared between exposure groups using the log-rank test. Cox proportional hazards regression models were applied to estimate hazard ratios (HRs) and corresponding 95% confidence intervals (CIs) for all clinical outcomes.

The proportional hazards assumption was formally evaluated. Within the TriNetX analytic environment, after excluding patients who experienced the outcome before the prespecified time window, proportionality tests yielded *p*-values of 0.5401 for all-cause mortality, 0.6216 for MACE, 0.9346 for MAKE, and 0.8266 for fracture, indicating no evidence of violation of the proportional hazards assumption.

Missing data were handled using an available-case approach without imputation. Given the multiple outcomes and subgroup analyses performed, the results were interpreted as exploratory and no formal adjustment for multiple comparisons was applied. All statistical tests were two-sided, and a *p*-value < 0.05 was considered statistically significant. All analyses were performed within the secure TriNetX analytic environment using its standardized statistical pipelines.

### 2.7. Sensitivity Analyses

To evaluate the robustness of the primary findings, three prespecified sensitivity analyses were performed. First, landmark analyses at 1, 3, and 5 years were conducted to assess the temporal stability of the associations between VDD and clinical outcomes (Supplementary Table S2). Second, severity analyses were performed using alternative vitamin D exposure thresholds to examine graded associations between lower serum 25-hydroxyvitamin D concentrations and adverse outcomes (Supplementary Table S3). Third, E-value analyses were conducted to quantify the minimum strength of association that an unmeasured confounder would need to have with both vitamin D status and each outcome to fully explain away the observed associations (Supplementary Table S4).

## 3. Results

### 3.1. Patient Identification, Baseline Characteristics, and Propensity Score Matching

A total of 19,177 adult patients with a diagnosis of SjS and osteoporosis were identified from the TriNetX cohort between 1 January 2010 and 1 January 2024. Among these patients, 1236 individuals had serum 25-hydroxyvitamin D concentrations below 20 ng/mL and were classified as having VDD, while 7753 individuals had concentrations of 30 ng/mL or higher and were classified as having VDA.

**Figure 1:**
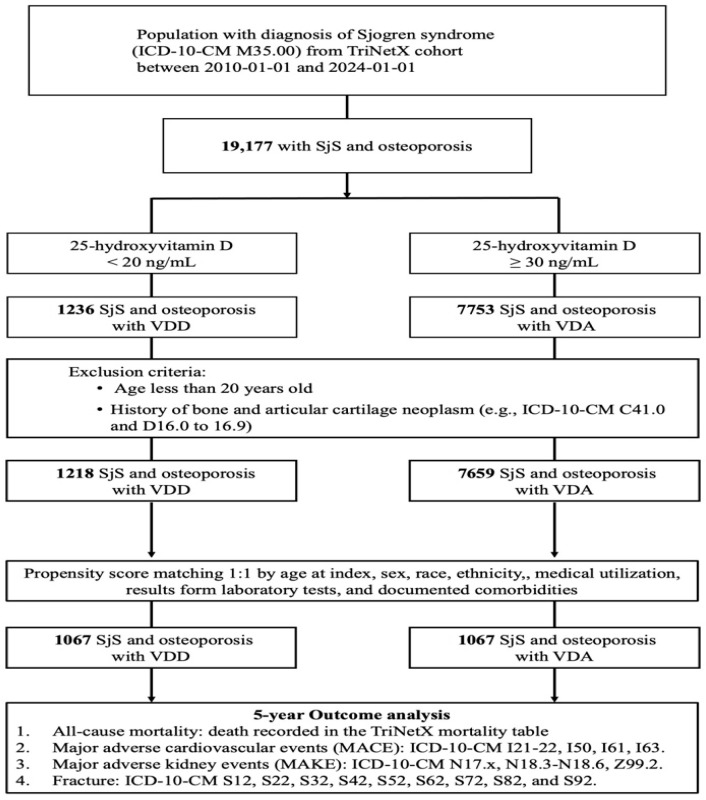
Algorithm for patient selection and enrollment in the study. Abbreviations: SjS, Sjögren syndrome; VDD, vitamin D deficiency; VDA, vitamin D adequacy; MACEs, major adverse cardiovascular events; MAKEs, major adverse kidney events.

After excluding patients younger than 20 years and those with a history of bone or articular cartilage neoplasms, 1218 patients remained in the VDD group and 7659 patients in the VDA group. These patients constituted the eligible source population for propensity score matching. Using 1: 1 propensity score matching based on age at index, sex, race, ethnicity, healthcare utilization, laboratory test results, and documented comorbidities, 1067 well-balanced pairs were generated for the final analytic cohort (Figure 1).

Before matching, patients in the VDD group were older and had a higher burden of cardiometabolic comorbidities, renal dysfunction, inflammatory markers, and systemic steroid use. After matching, baseline demographic characteristics, comorbid conditions, medication use, laboratory parameters, and healthcare utilization metrics were well balanced between the two groups, with standardized mean differences below 0.1 for all covariates (Table 1). The full set of baseline covariates used for propensity score matching, including detailed comorbidities, medication use, laboratory values, and healthcare utilization metrics, is provided in Supplementary Table S1. The matched cohorts were subsequently followed for up to five years to evaluate the incidence of all-cause mortality, MACEs, MAKEs, and fractures. The matched cohorts were subsequently followed for up to five years to evaluate the incidence of all-cause mortality, MACEs, MAKEs, and fractures.

### 3.2. Primary Outcomes: Kaplan–Meier Survival Analysis

**Figure 2:**
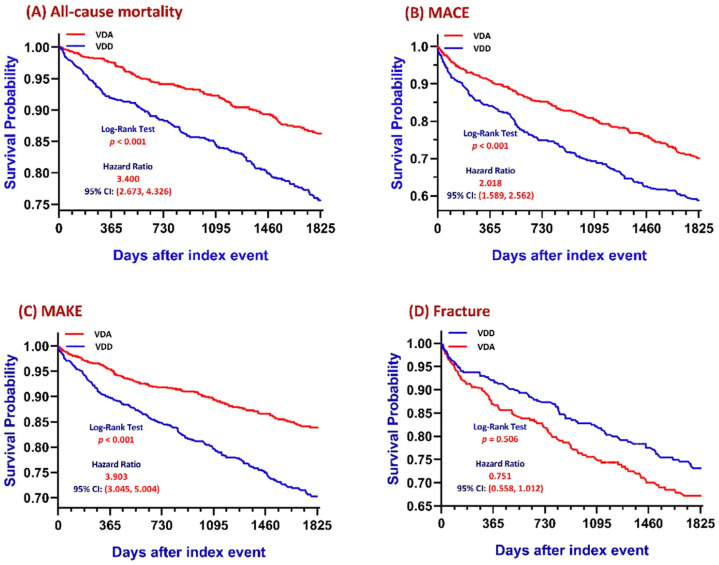
Kaplan–Meier Survival Curves for Primary Outcomes According to Vitamin D Status. Kaplan–Meier survival analyses comparing long-term clinical outcomes between patients with Sjögren’s syndrome and osteoporosis with vitamin D deficiency (VDD) and those with vitamin D adequacy (VDA) over a five-year follow-up period. (**A**) All-cause mortality. (**B**) MACE. (**C**) MAKE. (**D**) Fractures. Abbreviations: CI, confidence interval; MACE, major adverse cardiovascular events; MAKEs, major adverse kidney events.

Kaplan–Meier analyses demonstrated significant differences in long-term clinical outcomes between SjS patients with osteoporosis and VDD compared with those with VDA over the five-year follow-up period (Figure 2).

Patients with VDD exhibited a significantly lower survival probability and a higher risk of all-cause mortality compared with those with VDA (log-rank *p* < 0.001; Figure 2A). Similarly, event-free survival for MACE was significantly lower in the VDD group than in the VDA group (log-rank *p* < 0.001; Figure 2B). A comparable pattern was observed for MAKE, with VDD patients demonstrating significantly lower event-free survival over time (log-rank *p* < 0.001; Figure 2C). In contrast, no statistically significant difference was observed in fracture risk between the two groups during follow-up (log-rank *p* = 0.506; Figure 2D), indicating that VDD was not associated with an increased fracture risk in this cohort.

### 3.3. Subgroup and Effect Modification Analyses

Subgroup analyses were conducted to explore potential effect modification by key demographic and clinical characteristics. Across all outcomes, systemic comorbidities and organ involvement were more strongly associated with prognosis than SjS–specific manifestations.

**Figure 3:**
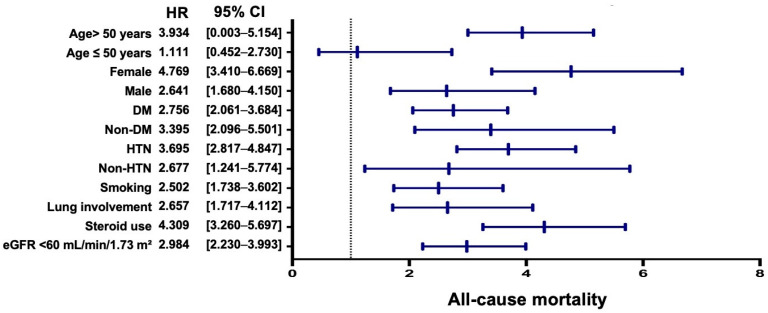
Subgroup Analysis of the Association Between Vitamin D Deficiency and All-Cause Mortality. Forest plot depicting subgroup analyses of the association between vitamin D deficiency and all-cause mortality. The dotted vertical line indicates the hazard ratio of one. Abbreviations: HR, hazard ratio; CI, confidence interval; DM, diabetes mellitus; HTN, hypertension; eGFR, estimated glomerular filtration rate.

For all-cause mortality, older age (>50 years), female sex, diabetes mellitus (DM), hypertension, smoking history, pulmonary involvement, systemic steroid use, and reduced baseline renal function (eGFR < 60 mL/min/1.73 m^2^) were each associated with a significantly increased risk, whereas age ≤ 50 years was not (Figure 3). Among these factors, systemic steroid use, advanced age, and female sex showed the largest effect estimates.

**Figure 4:**
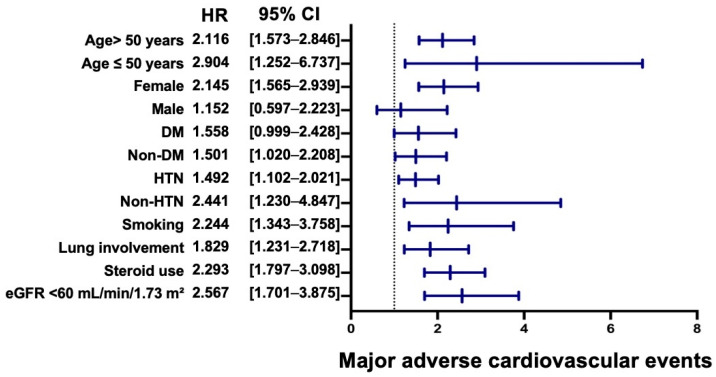
Subgroup Analysis of the Association Between Vitamin D Deficiency and Major Adverse Cardiovascular Events. Forest plot showing subgroup analyses of the association between vitamin D deficiency and major adverse cardiovascular events. The dotted vertical line indicates the hazard ratio of one. Abbreviations: HR, hazard ratio; CI, confidence interval; DM, diabetes mellitus; HTN, hypertension; eGFR, estimated glomerular filtration rate.

For MACE, older age, hypertension, smoking, pulmonary involvement, systemic steroid use, and impaired renal function were consistently associated with elevated cardiovascular risk (Figure 4). DM showed only a borderline association, and male sex was not significantly associated with MACE. The magnitude of association was greatest among patients with impaired renal function and those receiving systemic corticosteroid therapy.

**Figure 5:**
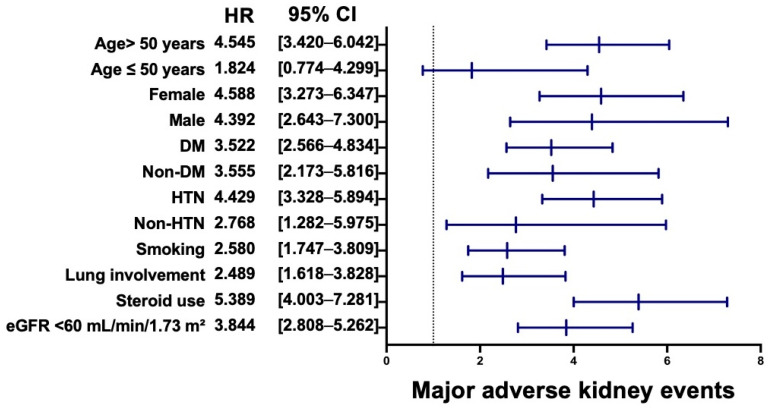
Subgroup Analysis of the Association Between Vitamin D Deficiency and Major Adverse Kidney Events. Forest plot showing subgroup analyses of the association between vitamin D deficiency and major adverse kidney events. The dotted vertical line indicates the hazard ratio of one. Abbreviations: HR, hazard ratio; CI, confidence interval; DM, diabetes mellitus; HTN, hypertension; eGFR, estimated glomerular filtration rate.

For MAKEs, older age, DM, hypertension, smoking, pulmonary involvement, systemic steroid use, and baseline renal impairment were again strongly associated with adverse renal outcomes, whereas age ≤ 50 years was not (Figure 5). Systemic steroid use, female sex, advanced age, and reduced baseline eGFR were associated with the highest risks of MAKEs.

Across all three outcomes, older age, hypertension, smoking, pulmonary involvement, systemic steroid use, and impaired renal function were consistently associated with increased risks, indicating that systemic comorbidities and organ involvement are major contributors to long-term prognosis in patients with SjS.

### 3.4. Sensitivity Analyses

To evaluate the robustness of the primary findings, three prespecified sensitivity analyses were performed, as summarized in Supplementary Tables S2–S5.

First, landmark analyses at 1, 3, and 5 years were conducted to assess the temporal stability of the associations between VDD and clinical outcomes. Across all landmark windows, patients with VDD consistently exhibited higher risks of all-cause mortality, MACE, and MAKE compared with those with VDA, whereas fracture risk remained comparable between groups (Supplementary Table S2).

Second, severity analyses were performed to examine a potential dose–response relationship across vitamin D categories. Compared with patients with VDA, those with VDD demonstrated significantly higher risks of MACE and MAKE, with a similar trend observed for all-cause mortality. Patients with vitamin D insufficiency, defined as serum 25-hydroxyvitamin D concentrations between 20 and 29 ng/mL, exhibited intermediate risk estimates between the deficiency and adequacy groups, supporting a graded association between lower vitamin D levels and adverse cardiorenal outcomes. No significant association was observed between vitamin D status and fracture risk across severity categories (Supplementary Table S3).

Third, E-value analyses were conducted to quantify the minimum strength of association that an unmeasured confounder would need to have with both vitamin D status and each outcome, on the risk ratio scale, to fully explain away the observed associations. The observed associations for MACE and MAKE were moderately robust to potential unmeasured confounding, whereas the E-value for all-cause mortality suggested modest sensitivity to residual confounding. The null association between VDD and fracture risk was consistent with the corresponding E-value estimates (Supplementary Table S4).

In addition, longitudinal analyses of vitamin D exposure were performed to examine the persistence of vitamin D status during follow-up (Supplementary Table S5). Patients classified as having VDD at baseline consistently demonstrated substantially lower mean serum 25-hydroxyvitamin D levels than those in the VDA group during both early (0–3 years) and late (4–5 years) follow-up periods. The frequency of documented vitamin D deficiency diagnoses was higher in the VDD group during early follow-up, whereas no significant difference was observed during late follow-up.

## 4. Discussion

This large-scale real-world cohort study shows that VDD is associated with significantly increased risks of all-cause mortality, major adverse cardiovascular events, and major adverse kidney events among patients with SjS and osteoporosis. After rigorous propensity score matching and multiple sensitivity analyses, patients with serum 25-hydroxyvitamin D concentrations < 20 ng/mL consistently exhibited poorer long-term outcomes compared with those with VDA. In contrast, fracture risk did not differ significantly between groups, supporting the interpretation that VDD in this population primarily reflects systemic disease burden and clinical vulnerability rather than skeletal vulnerability alone.

The association between VDD and increased all-cause mortality in SjS patients with osteoporosis highlights the potential relevance of vitamin D-related pathways in immune regulation, systemic inflammation, and organ protection. VDD has been shown to reduce vitamin D receptor activation and to shift immune balance toward a pro-inflammatory phenotype characterized by increased Th1 and Th17 activity and impaired regulatory T-cell function. This immune dysregulation has been associated with increased production of pro-inflammatory cytokines such as interleukin-6, tumor necrosis factor-α, and interleukin-17, which contribute to endothelial dysfunction, oxidative stress, and multisystem injury [23]. In autoimmune diseases, including SjS, chronic inflammation is a major driver of cardiovascular, renal, and metabolic complications, which together shape long-term survival. Our findings therefore suggest that VDD may serve as a marker of a high-risk clinical phenotype characterized by immune–metabolic dysregulation and greater disease burden.

Subgroup analyses further indicated that older age, DM, hypertension, smoking, pulmonary involvement, systemic corticosteroid use, and impaired renal function markedly amplified mortality risk. These findings are consistent with the concept that VDD acts as a vulnerability marker that interacts synergistically with traditional cardiometabolic and inflammatory risk factors. Female sex was also associated with higher mortality risk, which may reflect interactions between estrogen metabolism, immune activation, and vitamin D signaling [24]. Taken together, these observations support the interpretation that VDD in SjS reflects systemic frailty and immune–metabolic disturbance, rather than an isolated nutritional abnormality.

Cardiovascular outcomes were likewise strongly associated with vitamin D status. Patients with VDD experienced a significantly higher incidence of MACEs, including myocardial infarction, heart failure, ischemic stroke, and intracerebral hemorrhage. Mechanistically, VDD has been shown to disrupt the renin–angiotensin–aldosterone system, leading to increased angiotensin II activity, vascular remodeling, arterial stiffness, and cardiac hypertrophy [25]. In addition, VDD has been associated with endothelial dysfunction and accelerates atherosclerosis through enhanced inflammatory signaling, foam cell formation, and smooth muscle proliferation [26]. In SjS, chronic immune activation further amplifies vascular injury, creating a permissive environment for accelerated cardiovascular disease. Our subgroup analyses showed that advanced age, diabetes, hypertension, smoking, pulmonary involvement, corticosteroid use, and renal impairment were major contributors to cardiovascular risk, consistent with prior epidemiological studies. Importantly, the association between VDD and MACE persisted after accounting for these comorbidities, supporting its role as an indicator of overall cardiovascular vulnerability rather than a specific causal determinant.

The association between VDD and adverse renal outcomes was similarly pronounced. Patients with VDD exhibited a substantially higher risk of MAKEs, including acute kidney injury, progression to advanced chronic kidney disease, and dialysis dependence. VDD has been linked to renal injury through multiple pathways, including RAAS activation, intraglomerular hypertension, oxidative stress, and profibrotic signaling. Angiotensin II stimulates the expression of transforming growth factor-β and connective tissue growth factor, thereby accelerating extracellular matrix deposition and tubulointerstitial fibrosis [27,28]. These effects are further amplified by inflammatory cytokines and endothelial dysfunction [29]. In SjS, renal involvement may manifest as interstitial nephritis, glomerulonephritis, or tubular dysfunction, and VDD may exacerbate these processes by weakening immunoregulatory and antifibrotic mechanisms. Subgroup analyses indicated that diabetes, hypertension, smoking, pulmonary involvement, corticosteroid exposure, and baseline renal impairment were associated with increased MAKE risk, underscoring the multifactorial nature of renal vulnerability in this population.

Systemic corticosteroid therapy, commonly used to manage extraglandular manifestations of SjS, emerged as a particularly strong predictor of both cardiovascular and renal events. Prior studies have shown that corticosteroids increase cardiovascular risk in primary SjS, with Wu et al. reporting a 45% increase in coronary heart disease risk among steroid-treated patients. Corticosteroids also promote oxidative stress, endothelial dysfunction, and profibrotic signaling, thereby accelerating renal injury through upregulation of TGF-β1 and inflammatory mediators [30,31,32]. In the context of VDD, these adverse effects may be further potentiated, highlighting the importance of careful risk–benefit assessment and close monitoring in steroid-treated SjS patients.

In contrast to its strong associations with mortality and cardiorenal outcomes, VDD was not associated with an increased risk of fractures in this cohort of SjS patients with osteoporosis. This finding reflects the complex and multifactorial pathogenesis of bone fragility in autoimmune diseases, which involves chronic inflammation, immune-mediated osteoclast activation, hormonal changes, reduced physical activity, and long-term glucocorticoid exposure [6,33,34]. In this context, skeletal outcomes may be more strongly influenced by disease-related and treatment-related factors than by vitamin D status alone. Furthermore, the widespread use of anti-osteoporotic therapies and immunomodulatory agents such as hydroxychloroquine, which has been shown to inhibit osteoclastogenesis and preserve bone mineral density [35], may have attenuated the effect of VDD on fracture risk. These findings further support the view that in SjS patients with established osteoporosis, VDD primarily reflects systemic disease severity rather than skeletal fragility per se.

This study has several limitations. First, the retrospective observational design precludes causal inference and is subject to residual confounding despite extensive propensity score matching and sensitivity analyses. Second, heterogeneity in treatment regimens, including corticosteroids, immunosuppressive agents, and anti-osteoporotic therapies, may have influenced outcomes. Third, detailed measures of SjS disease activity, duration, and organ-specific severity were not uniformly available in the database. In addition, data on dietary intake, physical activity, sunlight exposure, and adherence to vitamin D supplementation were lacking. Furthermore, vitamin D testing in routine clinical practice is not randomly performed and may preferentially occur in patients with greater comorbidity burden, frailty, or higher healthcare utilization; therefore, vitamin D deficiency may partly reflect a higher-risk clinical phenotype rather than an isolated nutritional exposure. Moreover, the use of an available-case approach for missing data may have introduced additional selection bias. Finally, the study population was derived from a large healthcare network and may not be fully representative of all SjS populations worldwide. Accordingly, the present findings should be interpreted as demonstrating an association between VDD and adverse outcomes, rather than evidence of a causal or independent prognostic effect. Prospective studies are needed to clarify whether correction of VDD modifies long-term cardiorenal risk or survival in this vulnerable population.

## 5. Conclusions

In this large real-world cohort of patients with SjS and osteoporosis, VDD was associated with increased risks of all-cause mortality, MACEs, and MAKEs. These associations remained robust after extensive propensity score matching and sensitivity analyses. In contrast, fracture risk did not differ significantly according to vitamin D status. Our findings indicate that VDD is best interpreted as a marker of a high-risk systemic clinical phenotype rather than a determinant of skeletal vulnerability alone. Assessment of vitamin D status may therefore be useful for identifying patients at increased risk of adverse cardiorenal outcomes and for informing clinical risk stratification. However, causal inferences and therapeutic implications cannot be established from this observational study.

## Figures and Tables

**Table 1:** Key baseline characteristics of vitamin D deficient (VDD) and vitamin D adequate (VDA) groups before and after propensity score matching.

Characteristics	Before Matching—VDD vs. VDA				After Matching—VDD vs. VDA			
	Mean ± SD	Patient Count	% of Cohort	Standardized Difference	Mean ± SD	Patient Count	% of Cohort	Standardized Difference
Demographics								
Age at Index	55.59 ± 14.48 66.97 ± 10.73	908 5536	100.00% 100.00%	0.893	59.23 ± 12.74 59.35 ± 14.64	648 648	100.00% 100.00%	0.008
Female		670 5124	73.79% 92.56%	0.518		519 529	80.09% 81.64%	0.039
Diagnosis								
Hypertensive diseases		604 2759	66.52% 49.84%	0.343		394 392	60.80% 60.49%	0.006
Diabetes mellitus		432 972	47.58% 17.56%	0.676		249 258	38.43% 39.81%	0.028
Cerebrovascular diseases		101 394	11.12% 7.12%	0.140		65 54	10.03% 8.33%	0.058
Medications								
Prednisone		331 1337	36.45% 24.15%	0.270		224 210	34.57% 32.41%	0.045
ACE inhibitors		232 684	25.55% 12.36%	0.341		139 133	21.45% 20.52%	0.022
Angiotensin II inhibitors		132 770	14.54% 13.91%	0.018		88 88	13.58% 13.58%	< 0.001
Laboratory results								
Calcium	9.01 ± 0.85 9.40 ± 0.59	803 4367	88.44% 78.88%	0.538	9.11 ± 0.83 9.25 ± 0.69	550 523	84.88% 80.71%	0.176
(6–7 mg/dL)		81 88	8.92% 1.59%	0.333		34 31	5.25% 4.78%	0.021
(7–7.5 mg/dL)		154 161	16.96% 2.91%	0.483		68 64	10.49% 9.88%	0.020
(7.5–8 mg/dL)		265 343	29.18% 6.20%	0.631		125 115	19.29% 17.75%	0.039
(8–8.5 mg/dL)		376 592	41.41% 10.69%	0.747		193 175	29.78% 27.01%	0.061
(8.5–9 mg/dL)		499 1326	54.96% 23.95%	0.668		283 258	43.67% 39.81%	0.078
(9–9.5 mg/dL)		562 2629	61.89% 47.49%	0.292		361 349	55.71% 53.86%	0.037
(9.5–10 mg/dL)		420 2536	46.26% 45.81%	0.009		286 300	44.14% 46.30%	0.043
(10–10.5 mg/dL)		181 996	19.93% 17.99%	0.049		130 127	20.06% 19.60%	0.011
(10.5–11 mg/dL)		54 253	5.95% 4.57%	0.061		34 34	5.25% 5.25%	< 0.001
(11–11.5 mg/dL)		14 54	1.54% 0.97%	0.050		11 10	1.70% 1.54%	0.012
(11.5–12 mg/dL)		10 17	1.10% 0.31%	0.095		10 10	1.54% 1.54%	< 0.001
Creatinine	2.36 ± 7.06 1.44 ± 7.98	796 4445	87.67% 80.29%	0.123	2.22 ± 8.35 1.65 ± 6.30	545 532	84.11% 82.10%	0.076
HbA1c	7.32 ± 2.11 6.14 ± 1.28	444 1637	48.90% 29.57%	0.677	6.84 ± 1.68 6.92 ± 1.83	269 258	41.51% 39.81%	0.046
(4–5%)		32 94	3.52% 1.70%	0.114		17 23	2.62% 3.55%	0.053
(5–6%)		161 1015	17.73% 18.34%	0.015		114 107	17.59% 16.51%	0.028
(6–7%)		138 561	15.20% 10.13%	0.152		98 86	15.12% 13.27%	0.053
(7–8%)		117 257	12.88% 4.64%	0.294		70 73	10.80% 11.27%	0.014
(8–9%)		83 126	9.14% 2.28%	0.299		47 46	7.25% 7.10%	0.006
(9–11%)		92 73	10.13% 1.32%	0.386		34 41	5.25% 6.33%	0.046
(11–13%)		48 17	5.29% 0.31%	0.305		10 14	1.54% 2.16%	0.045
Calcidiol	11.94 ± 4.61 49.98 ± 16.66	198 1353	21.81% 24.44%	3.112	12.55 ± 4.54 47.25 ± 14.35	135 135	20.83% 20.83%	3.262

Abbreviations: HbA1c, hemoglobin A1c; LDL, low-density lipoprotein; SD, standard deviation; Std. Diff., standardized difference; VDA, vitamin D adequacy; VDD, vitamin D deficiency.

## Data Availability

The data presented in this study are available on request from the corresponding author due to privacy and ethical restrictions. The dataset was obtained from the TriNetX global federated health research network, which aggregates deidentified electronic medical records from multiple healthcare institutions. Access to the dataset is restricted by institutional policies and data-sharing agreements. Researchers interested in accessing the data may request it from TriNetX, subject to institutional approval and compliance with data privacy regulations.

## Supplementary Materials

The following supporting information can be downloaded at: https://www.mdpi.com/article/10.3390/jcm15041430/s1, Table S1: Baseline characteristics of vitamin D deficient (VDD) and vitamin D adequate (VDA) groups before and after propensity score matching; Table S2: Landmark 1-, 3-, and 5-Year Kaplan–Meier Survival Analyses in Patients with Sjögren’s Syndrome and Osteoporosis According to Vitamin D Status; Table S3: Severity of Vitamin D Deficiency and 5-Year Kaplan–Meier Outcomes in Patients with Sjögren’s Syndrome and Osteoporosis; Table S4: E-value Sensitivity Analysis for Primary Outcomes According to Vitamin D Status in Patients with Sjögren’s Syndrome and Osteoporosis; Table S5: Longitudinal vitamin D exposure in patients with Sjögren’s syndrome and osteoporosis.

## Abbreviations

The following abbreviations are used in this manuscript:

25(OH)D	25-Hydroxyvitamin D
ACEI	Angiotensin-Converting Enzyme Inhibitor
ARB	Angiotensin II Receptor Blocker
CKD	Chronic Kidney Disease
CRP	C-Reactive Protein
DM	Diabetes Mellitus
eGFR	Estimated Glomerular Filtration Rate
HbA1c	Hemoglobin A1c
HDL	High-Density Lipoprotein
LDL	Low-Density Lipoprotein
MACE	Major Adverse Cardiovascular Events
MAKE	Major Adverse Kidney Events
RAAS	Renin–Angiotensin–Aldosterone System
SjS	Sjögren’s Syndrome
VDA	Vitamin D Adequacy
VDD	Vitamin D Deficiency
