# Supplementary material for: Association of Vitamin D Deficiency with Mortality and Cardiorenal Events in Sjögren’s Syndrome and Osteoporosis
Source: J Clin Med. 2026 Feb 12;15(4):1430. doi: 10.3390/jcm15041430 (PMC12942526; doi:10.3390/jcm15041430)
Supplement: Supplementary file 1 [file jcm-15-01430-s001.zip › jcm-4121286-supplementary.pdf]

Supplementary materials

Table S1. Baseline characteristics of vitamin D deficient (VDD) and vitamin D adequate (VDA) groups before and after propensity score matching.

Table S2. Landmark 1-, 3-, and 5-Year Kaplan–Meier Survival Analyses in Patients with Sjögren's Syndrome and Osteoporosis According to Vitamin D Status.

Table S3. Severity of Vitamin D Deficiency and 5-Year Kaplan–Meier Outcomes in Patients with Sjögren's Syndrome and Osteoporosis.

Table S4. E-value Sensitivity Analysis for Primary Outcomes According to Vitamin D Status in Patients with Sjögren's Syndrome and Osteoporosis.

Table S5. Longitudinal vitamin D exposure in patients with Sjögren's syndrome and osteoporosis.

Supplementary Table S1. Baseline characteristics of vitamin D deficient (VDD) and vitamin D adequate (VDA) groups before and after propensity score matching.

Characteristics	Before Matching – VDD vs. VDA				After Matching – VDD vs. VDA			
	Mean±SD	Patient Count	% of Cohort	Standardized Difference	Mean±SD	Patient Count	% of Cohort	Standardized Difference
Demographics								
Age at Index	55.59±14.48<br>66.97±10.73	908<br>5,536	100.00%<br>100.00%	0.893	59.23±12.74<br>59.35±14.64	648<br>648	100.00%<br>100.00%	0.008
Female		670<br>5,124	73.79%<br>92.56%	0.518		519<br>529	80.09%<br>81.64%	0.039
White		447<br>4,292	49.23%<br>77.53%	0.615		378<br>366	58.33%<br>56.48%	0.037
Black or African American		301<br>417	33.15%<br>7.53%	0.671		162<br>166	25.00%<br>25.62%	0.014
Asian		16<br>361	1.76%<br>6.52%	0.241		16<br>16	2.47%<br>2.47%	<0.001
Diagnosis								
Hypertensive diseases		604<br>2,759	66.52%<br>49.84%	0.343		394<br>392	60.80%<br>60.49%	0.006
Systemic connective tissue disorders		376<br>4,026	41.41%<br>72.72%	0.667		328<br>314	50.62%<br>48.46%	0.043
Diabetes mellitus		432	47.58%	0.676		249	38.43%	0.028

		972	17.56%			258	39.81%	
Osteoarthritis		215<br>2,013	23.68%<br>36.36%	0.279		185<br>190	28.55%<br>29.32%	0.017
Inflammatory polyarthropathies		202<br>1,428	22.25%<br>25.80%	0.083		156<br>160	24.07%<br>24.69%	0.014
Overweight, obesity and other hyperalimentation		239<br>705	26.32%<br>12.73%	0.348		150<br>159	23.15%<br>24.54%	0.032
Ischemic heart diseases		228<br>759	25.11%<br>13.71%	0.291		138<br>130	21.30%<br>20.06%	0.030
Cerebrovascular diseases		101<br>394	11.12%<br>7.12%	0.140		65<br>54	10.03%<br>8.33%	0.058
Medications								
Diuretics		448<br>1,321	49.34%<br>23.86%	0.548		280<br>267	43.21%<br>41.20%	0.040
Blood glucose regulation agents		461<br>1,073	50.77%<br>19.38%	0.696		260<br>251	40.12%<br>38.73%	0.028
Beta blockers		411<br>1,397	45.26%<br>25.23%	0.428		258<br>241	39.81%<br>37.19%	0.053
Prednisone		331<br>1,337	36.45%<br>24.15%	0.270		224<br>210	34.57%<br>32.41%	0.045
Calcium channel blockers		336<br>1,155	37.00%<br>20.86%	0.361		211<br>195	32.56%<br>30.09%	0.053

ACE inhibitors		232<br>684	25.55%<br>12.36%	0.341		139<br>133	21.45%<br>20.52%	0.022
Angiotensin II inhibitors		132<br>770	14.54%<br>13.91%	0.018		88<br>88	13.58%<br>13.58%	< 0.001
Laboratory results								
Potassium	4.23±0.65<br>4.19±0.44	819<br>4,399	90.20%<br>79.46%	0.071	4.19±0.62<br>4.21±0.50	561<br>553	86.57%<br>85.34%	0.022
Sodium	138.08±4.18<br>138.91±3.17	813<br>4,389	89.54%<br>79.28%	0.224	138.41±4.35<br>138.46±3.65	557<br>547	85.96%<br>84.41%	0.010
(120-125 mmol/L)		38<br>51	4.18%<br>0.92%	0.208		14<br>14	2.16%<br>2.16%	< 0.001
(125-130 mmol/L)		122<br>230	13.44%<br>4.16%	0.332		61<br>62	9.41%<br>9.57%	0.005
(130-135 mmol/L)		362<br>865	39.87%<br>15.62%	0.562		189<br>184	29.17%<br>28.39%	0.017
(135-140 mmol/L)		670<br>2,881	73.79%<br>52.04%	0.462		434<br>415	66.97%<br>64.04%	0.061
(140-145 mmol/L)		578<br>2,884	63.66%<br>52.09%	0.235		380<br>375	58.64%<br>57.87%	0.015
Bicarbonate	25.30±4.36<br>26.75±3.25	802<br>4,378	88.33%<br>79.08%	0.378	25.70±4.14<br>26.10±3.41	551<br>542	85.03%<br>83.64%	0.102
(15-18 mmol/L)		158	17.40%	0.497		69	10.65%	0.073

		157	2.84%			55	8.49%	
(18-20 mmol/L)		219<br>311	24.12%<br>5.62%	0.538		106<br>94	16.36%<br>14.51%	0.051
(20-22 mmol/L)		314<br>621	34.58%<br>11.22%	0.578		161<br>161	24.85%<br>24.85%	< 0.001
(22-24 mmol/L)		427<br>1,087	47.03%<br>19.64%	0.607		241<br>241	37.19%<br>37.19%	< 0.001
(24-26 mmol/L)		493<br>1,789	54.30%<br>32.32%	0.454		292<br>314	45.06%<br>48.46%	0.068
Calcium	9.01±0.85<br>9.40±0.59	803<br>4,367	88.44%<br>78.88%	0.538	9.11±0.83<br>9.25±0.69	550<br>523	84.88%<br>80.71%	0.176
(6-7 mg/dL)		81<br>88	8.92%<br>1.59%	0.333		34<br>31	5.25%<br>4.78%	0.021
(7-7.5 mg/dL)		154<br>161	16.96%<br>2.91%	0.483		68<br>64	10.49%<br>9.88%	0.020
(7.5-8 mg/dL)		265<br>343	29.18%<br>6.20%	0.631		125<br>115	19.29%<br>17.75%	0.039
(8-8.5 mg/dL)		376<br>592	41.41%<br>10.69%	0.747		193<br>175	29.78%<br>27.01%	0.061
(8.5-9 mg/dL)		499<br>1,326	54.96%<br>23.95%	0.668		283<br>258	43.67%<br>39.81%	0.078
(9-9.5 mg/dL)		562	61.89%	0.292		361	55.71%	0.037

		2,629	47.49%			349	53.86%	
(9.5-10 mg/dL)		420<br>2,536	46.26%<br>45.81%	0.009		286<br>300	44.14%<br>46.30%	0.043
(10-10.5 mg/dL)		181<br>996	19.93%<br>17.99%	0.049		130<br>127	20.06%<br>19.60%	0.011
(10.5-11 mg/dL)		54<br>253	5.95%<br>4.57%	0.061		34<br>34	5.25%<br>5.25%	< 0.001
(11-11.5 mg/dL)		14<br>54	1.54%<br>0.97%	0.050		11<br>10	1.70%<br>1.54%	0.012
(11.5-12 mg/dL)		10<br>17	1.10%<br>0.31%	0.095		10<br>10	1.54%<br>1.54%	< 0.001
Creatinine	2.36±7.06<br>1.44±7.98	796<br>4,445	87.67%<br>80.29%	0.123	2.22±8.35<br>1.65±6.30	545<br>532	84.11%<br>82.10%	0.076
Urea nitrogen	26.14±20.11<br>18.59±10.45	774<br>4,381	85.24%<br>79.14%	0.471	23.66±18.43<br>21.78±15.38	536<br>523	82.72%<br>80.71%	0.110
(10-14 mg/dL)		339<br>1,736	37.34%<br>31.36%	0.126		236<br>230	36.42%<br>35.49%	0.019
(14-18 mg/dL)		382<br>2,162	42.07%<br>39.05%	0.061		259<br>254	39.97%<br>39.20%	0.015
(18-22 mg/dL)		325<br>1,711	35.79%<br>30.91%	0.103		213<br>197	32.87%<br>30.40%	0.053
(22-26 mg/dL)		265	29.18%	0.217		159	24.54%	< 0.001

		1,101	19.89%			159	24.54%	
(26-30 mg/dL)		256<br>712	28.19%<br>12.86%	0.386		142<br>144	21.91%<br>22.22%	0.007
(30-35 mg/dL)		241<br>485	26.54%<br>8.76%	0.479		123<br>124	18.98%<br>19.14%	0.004
(35-40 mg/dL)		239<br>342	26.32%<br>6.18%	0.567		116<br>112	17.90%<br>17.28%	0.016
(40-45 mg/dL)		199<br>239	21.92%<br>4.32%	0.540		94<br>90	14.51%<br>13.89%	0.017
(45-50 mg/dL)		185<br>197	20.37%<br>3.56%	0.536		85<br>81	13.12%<br>12.50%	0.018
(50-55 mg/dL)		200<br>207	18.85%<br>3.07%	0.5221		103<br>91	13.37%<br>11.81%	0.0470
(55-60 mg/dL)		156<br>121	17.18%<br>2.19%	0.524		69<br>67	10.65%<br>10.34%	0.010
Erythrocytes	3.88±0.84<br>4.11±0.84	788<br>4,287	86.78%<br>77.44%	0.283	4.00±0.81<br>3.97±0.93	533<br>524	82.25%<br>80.86%	0.033
Hemoglobin	11.25±2.33<br>12.65±1.68	784<br>4,071	86.34%<br>73.54%	0.693	11.67±2.25<br>11.92±2.09	530<br>516	81.79%<br>79.63%	0.114
(6-8 g/dL)		232<br>236	25.55%<br>4.26%	0.626		103<br>96	15.89%<br>14.81%	0.030
(8-9 g/dL)		287	31.61%	0.680		137	21.14%	0.034

		351	6.34%			128	19.75%	
(9-10 g/dL)		340<br>552	37.45%<br>9.97%	0.682		181<br>168	27.93%<br>25.93%	0.045
(10-11 g/dL)		405<br>829	44.60%<br>14.97%	0.684		220<br>208	33.95%<br>32.10%	0.039
(11-12 g/dL)		391<br>1,228	43.06%<br>22.18%	0.456		242<br>227	37.35%<br>35.03%	0.048
(12-13 g/dL)		338<br>1,762	37.23%<br>31.83%	0.113		242<br>235	37.35%<br>36.27%	0.022
(13-14 g/dL)		282<br>1,779	31.06%<br>32.13%	0.023		212<br>198	32.72%<br>30.56%	0.046
(14-15 g/dL)		150<br>1,051	16.52%<br>18.98%	0.064		116<br>120	17.90%<br>18.52%	0.016
Alanine aminotransferase	25.47±33.04<br>22.23±14.68	754<br>4,147	83.04%<br>74.91%	0.126	24.70±20.86<br>25.04±21.86	512<br>504	79.01%<br>77.78%	0.016
Aspartate aminotransferase	29.50±35.20<br>24.96±12.50	755<br>4,136	83.15%<br>74.71%	0.171	27.16±22.35<br>26.56±16.77	512<br>502	79.01%<br>77.47%	0.030
(30-35 U/L)		217<br>819	23.90%<br>14.79%	0.232		127<br>127	19.60%<br>19.60%	< 0.001
(35-40 U/L)		167<br>465	18.39%<br>8.40%	0.296		92<br>100	14.20%<br>15.43%	0.034
(40-45 U/L)		132	14.54%	0.306		64	9.88%	0.005

		302	5.46%			65	10.03%	
(45-50 U/L)		104<br>215	11.45%<br>3.88%	0.287		58<br>58	8.95%<br>8.95%	< 0.001
Leukocytes	14.50±181.48<br>9.90±117.14	757<br>3,913	83.37%<br>70.68%	0.030	17.45±221.10<br>16.82±209.91	510<br>501	78.70%<br>77.31%	0.003
Albumin	3.57±0.78<br>4.05±0.46	741<br>4,060	81.61%<br>73.34%	0.745	3.73±0.74<br>3.84±0.61	509<br>480	78.55%<br>74.07%	0.164
(2.5-3 g/dL)		252<br>325	27.75%<br>5.87%	0.611		117<br>116	18.06%<br>17.90%	0.004
(3-3.2 g/dL)		238<br>368	26.21%<br>6.65%	0.547		117<br>107	18.06%<br>16.51%	0.040
(3.2-3.4 g/dL)		262<br>528	28.86%<br>9.54%	0.505		145<br>130	22.38%<br>20.06%	0.056
(3.4-3.6 g/dL)		300<br>783	33.04%<br>14.14%	0.456		182<br>158	28.09%<br>24.38%	0.084
(3.6-3.8 g/dL)		328<br>1,216	36.12%<br>21.96%	0.315		211<br>198	32.56%<br>30.56%	0.043
(3.8-4 g/dL)		343<br>1,745	37.77%<br>31.52%	0.131		235<br>238	36.27%<br>36.73%	0.009
(4-4.2 g/dL)		253<br>1,570	27.86%<br>28.36%	0.011		174<br>177	26.85%<br>27.32%	0.010
(4.2-4.4 g/dL)		238	26.21%	0.185		182	28.09%	< 0.001

		1,921	34.70%			182	28.09%	
(4.4-4.6 g/dL)		133<br>1,126	14.65%<br>20.34%	0.150		105<br>112	16.20%<br>17.28%	0.029
(4.6-5 g/dL)		78<br>724	8.59%<br>13.08%	0.144		61<br>66	9.41%<br>10.19%	0.026
Alkaline phosphatase	117.22±114.76<br>78.95±37.93	744<br>3,972	81.94%<br>71.75%	0.447	103.69±90.10<br>97.59±53.68	502<br>486	77.47%<br>75.00%	0.082
(10-20 U/L)		10<br>10	1.10%<br>0.18%	0.115		10<br>10	1.54%<br>1.54%	< 0.001
(20-30 U/L)		10<br>42	1.10%<br>0.76%	0.035		10<br>10	1.54%<br>1.54%	< 0.001
(30-50 U/L)		79<br>734	8.70%<br>13.26%	0.146		61<br>51	9.41%<br>7.87%	0.054
(50-70 U/L)		260<br>1,772	28.63%<br>32.01%	0.073		181<br>173	27.93%<br>26.70%	0.027
(70-90 U/L)		333<br>1,593	36.67%<br>28.77%	0.168		222<br>231	34.26%<br>35.65%	0.029
(90-110 U/L)		303<br>1,000	33.37%<br>18.06%	0.355		178<br>178	27.47%<br>27.47%	< 0.001
(110-130 U/L)		232<br>499	25.55%<br>9.01%	0.448		127<br>125	19.60%<br>19.29%	0.008
(130-150 U/L)		178	19.60%	0.441		95	14.66%	0.022

		296	5.35%			100	15.43%	
(150-170 U/L)		126<br>166	13.88%<br>3.00%	0.399		64<br>67	9.88%<br>10.34%	0.015
(170-190 U/L)		82<br>110	9.03%<br>1.99%	0.312		40<br>40	6.17%<br>6.17%	< 0.001
(190-210 U/L)		59<br>80	6.50%<br>1.45%	0.260		27<br>28	4.17%<br>4.32%	0.008
(210-250 U/L)		70<br>86	7.71%<br>1.55%	0.296		31<br>31	4.78%<br>4.78%	< 0.001
(250-300 U/L)		50<br>53	5.51%<br>0.96%	0.259		17<br>21	2.62%<br>3.24%	0.037
Protein	6.89±1.20<br>6.99±0.69	723<br>3,831	79.63%<br>69.20%	0.110	6.93±1.24<br>6.96±0.88	486<br>453	75.00%<br>69.91%	0.027
(3-4 g/dL)		25<br>13	2.75%<br>0.23%	0.208		11<br>10	1.70%<br>1.54%	0.012
(4-5 g/dL)		85<br>116	9.36%<br>2.10%	0.316		45<br>47	6.94%<br>7.25%	0.012
(5-6 g/dL)		216<br>478	23.79%<br>8.63%	0.420		111<br>104	17.13%<br>16.05%	0.029
(6-7 g/dL)		442<br>2,443	48.68%<br>44.13%	0.091		277<br>271	42.75%<br>41.82%	0.019
(7-8 g/dL)		474	52.20%	0.177		309	47.69%	0.034

		2,400	43.35%			298	45.99%	
(8-9 g/dL)		184<br>458	20.26%<br>8.27%	0.348		99<br>95	15.28%<br>14.66%	0.017
(9-10 g/dL)		30<br>56	3.30%<br>1.01%	0.158		11<br>10	1.70%<br>1.54%	0.012
(10-12 g/dL)		10<br>10	1.10%<br>0.18%	0.115		10<br>10	1.54%<br>1.54%	< 0.001
Phosphate	3.97±1.38<br>3.53±0.78	497<br>1,222	54.74%<br>22.07%	0.395	3.88±1.30<br>3.67±0.89	285<br>248	43.98%<br>38.27%	0.188
(2-3 mg/dL)		235<br>508	25.88%<br>9.18%	0.450		132<br>118	20.37%<br>18.21%	0.055
(3-4 mg/dL)		377<br>951	41.52%<br>17.18%	0.554		213<br>203	32.87%<br>31.33%	0.033
(4-5 mg/dL)		325<br>528	35.79%<br>9.54%	0.660		169<br>157	26.08%<br>24.23%	0.043
(5-6 mg/dL)		195<br>136	21.48%<br>2.46%	0.612		85<br>76	13.12%<br>11.73%	0.042
(6-7 mg/dL)		110<br>58	12.12%<br>1.05%	0.457		42<br>38	6.48%<br>5.86%	0.026
Triglyceride	158.84±140.10<br>117.97±67.14	431<br>2,260	47.47%<br>40.82%	0.372	156.42±132.04<br>140.15±82.89	272<br>268	41.98%<br>41.36%	0.147
(100-120 mg/dL)		86	9.47%	0.054		53	8.18%	0.033

		439	7.93%			59	9.11%	
(120-140 mg/dL)		67<br>309	7.38%<br>5.58%	0.073		36<br>40	5.56%<br>6.17%	0.026
(140-160 mg/dL)		63<br>240	6.94%<br>4.33%	0.113		32<br>28	4.94%<br>4.32%	0.029
(160-180 mg/dL)		48<br>152	5.29%<br>2.75%	0.129		28<br>23	4.32%<br>3.55%	0.039
(180-200 mg/dL)		34<br>137	3.74%<br>2.48%	0.073		22<br>24	3.40%<br>3.70%	0.016
(200-220 mg/dL)		31<br>89	3.41%<br>1.61%	0.115		17<br>20	2.62%<br>3.09%	0.027
(220-240 mg/dL)		26<br>63	2.86%<br>1.14%	0.123		16<br>13	2.47%<br>2.01%	0.031
HbA1c	7.32±2.11<br>6.14±1.28	444<br>1,637	48.90%<br>29.57%	0.677	6.84±1.68<br>6.92±1.83	269<br>258	41.51%<br>39.81%	0.046
(4-5 %)		32<br>94	3.52%<br>1.70%	0.114		17<br>23	2.62%<br>3.55%	0.053
(5-6 %)		161<br>1,015	17.73%<br>18.34%	0.015		114<br>107	17.59%<br>16.51%	0.028
(6-7 %)		138<br>561	15.20%<br>10.13%	0.152		98<br>86	15.12%<br>13.27%	0.053
(7-8 %)		117	12.88%	0.294		70	10.80%	0.014

		257	4.64%			73	11.27%	
(8-9 %)		83<br>126	9.14%<br>2.28%	0.299		47<br>46	7.25%<br>7.10%	0.006
(9-11 %)		92<br>73	10.13%<br>1.32%	0.386		34<br>41	5.25%<br>6.33%	0.046
(11-13 %)		48<br>17	5.29%<br>0.31%	0.305		10<br>14	1.54%<br>2.16%	0.045
Cholesterol in LDL	96.48±44.11<br>96.48±36.43	413<br>2,260	45.48%<br>40.82%	< 0.001	99.80±43.48<br>97.06±45.13	264<br>269	40.74%<br>41.51%	0.061
(20-30 mg/dL)		11<br>27	1.21%<br>0.49%	0.078		10<br>10	1.54%<br>1.54%	< 0.001
(30-40 mg/dL)		17<br>49	1.87%<br>0.89%	0.084		10<br>11	1.54%<br>1.70%	0.012
(40-50 mg/dL)		34<br>115	3.74%<br>2.08%	0.099		16<br>17	2.47%<br>2.62%	0.010
(50-60 mg/dL)		42<br>173	4.63%<br>3.12%	0.077		27<br>34	4.17%<br>5.25%	0.051
(60-70 mg/dL)		50<br>268	5.51%<br>4.84%	0.030		34<br>35	5.25%<br>5.40%	0.007
(70-80 mg/dL)		45<br>312	4.96%<br>5.64%	0.030		28<br>29	4.32%<br>4.47%	0.008
(80-100 mg/dL)		111	12.22%	0.034		70	10.80%	< 0.001

		615	11.11%			70	10.80%	
(100-120 mg/dL)		77<br>529	8.48%<br>9.56%	0.037		47<br>44	7.25%<br>6.79%	0.018
(120-140 mg/dL)		65<br>352	7.16%<br>6.36%	0.031		42<br>45	6.48%<br>6.94%	0.019
(140-160 mg/dL)		39<br>203	4.29%<br>3.67%	0.032		29<br>32	4.47%<br>4.94%	0.022
Cholesterol in HDL	48.70±19.36<br>60.01±19.20	413<br>2,242	45.48%<br>40.50%	0.586	50.88±18.99<br>52.25±18.28	264<br>262	40.74%<br>40.43%	0.073
(20-30 mg/dL)		42<br>51	4.63%<br>0.92%	0.227		18<br>16	2.78%<br>2.47%	0.019
(30-40 mg/dL)		121<br>241	13.33%<br>4.35%	0.320		67<br>64	10.34%<br>9.88%	0.015
(40-50 mg/dL)		128<br>478	14.10%<br>8.63%	0.172		84<br>85	12.96%<br>13.12%	0.005
(50-60 mg/dL)		97<br>587	10.68%<br>10.60%	0.002		66<br>68	10.19%<br>10.49%	0.010
(60-70 mg/dL)		50<br>528	5.51%<br>9.54%	0.153		40<br>39	6.17%<br>6.02%	0.007
Cholesterol	175.32±61.62<br>179.60±45.08	417<br>2,236	45.92%<br>40.39%	0.079	179.52±62.84<br>177.72±53.58	263<br>257	40.59%<br>39.66%	0.031
(40-60 mg/dL)		0	0.00%	0.060		0	0.00%	0.177

		10	0.18%			10	1.54%	
(60-80 mg/dL)		10<br>10	1.10%<br>0.18%	0.115		10<br>10	1.54%<br>1.54%	< 0.001
(80-100 mg/dL)		34<br>34	3.74%<br>0.61%	0.215		13<br>12	2.01%<br>1.85%	0.011
(100-140 mg/dL)		93<br>370	10.24%<br>6.68%	0.128		51<br>57	7.87%<br>8.80%	0.034
(140-180 mg/dL)		164<br>845	18.06%<br>15.26%	0.075		103<br>104	15.89%<br>16.05%	0.004
(180-200 mg/dL)		72<br>482	7.93%<br>8.71%	0.028		45<br>42	6.94%<br>6.48%	0.019
(200-240 mg/dL)		87<br>622	9.58%<br>11.24%	0.054		56<br>55	8.64%<br>8.49%	0.006
(240-280 mg/dL)		47<br>180	5.18%<br>3.25%	0.095		32<br>37	4.94%<br>5.71%	0.034
(280-320 mg/dL)		20<br>48	2.20%<br>0.87%	0.108		10<br>10	1.54%<br>1.54%	< 0.001
Magnesium	1.89±0.32<br>1.97±0.28	430<br>1,228	47.36%<br>22.18%	0.282	1.89±0.32<br>1.90±0.31	252<br>232	38.89%<br>35.80%	0.039
Ferritin	652.65±1438.87<br>296.86±1831.95	337<br>925	37.12%<br>16.71%	0.216	514.19±937.46<br>701.04±4032.20	188<br>180	29.01%<br>27.78%	0.064
Calcidiol	11.94±4.61	198	21.81%	3.112	12.55±4.54	135	20.83%	3.262

	49.98±16.66	1,353	24.44%		47.25±14.35	135	20.83%	

Supplementary Table S2. Landmark 1-, 3-, and 5-Year Kaplan–Meier Survival Analyses in Patients with Sjögren's Syndrome and Osteoporosis According to Vitamin D Status. Landmark Kaplan–Meier survival analyses at 1, 3, and 5 years comparing patients with vitamin D deficiency (VDD; serum 25-hydroxyvitamin D <20 ng/mL) and vitamin D adequacy (VDA; ≥30 ng/mL). Outcomes include all-cause mortality, major adverse cardiovascular events (MACE), major adverse kidney events (MAKE), and fractures.

Follow Up	Outcomes	Cohorts	Patients in cohort	Patients with outcome	Survival probability	Hazard Ratio	95% CI	Log-Rank test P-value
1 year	All-cause mortality	VDD	964	107	88.41%	5.299	(3.349, 8.384)	< 0.001
		VDA	976	22	97.67%			
3 years		VDD	964	179	79.23%	3.312	(2.477, 4.430)	< 0.001
		VDA	976	61	92.70%			
5 years		VDD	1,028	269	67.54%	3.400	(2.673, 4.326)	< 0.001
		VDA	1,053	88	87.83%			
1 year	MACE	VDD	451	65	84.88%	2.230	(1.513, 3.287)	< 0.001

		VDA	599	42	92.74%			
3 years		VDD	451	119	69.54%	1.902	(1.452, 2.491)	< 0.001
		VDA	599	95	82.13%			
5 years		VDD	473	157	56.86%	2.018	(1.589, 2.562)	< 0.001
		VDA	649	118	75.69%			
1 year	MAKE	VDD	797	99	87.00%	3.982	(2.660, 5.961)	< 0.001
		VDA	921	31	96.52%			
3 years		VDD	797	172	75.52%	3.513	(2.636, 4.681)	< 0.001
		VDA	921	64	92.10%			
5 years		VDD	862	250	63.50%	3.903	(3.045, 5.004)	< 0.001
		VDA	989	83	87.73%			
1 years	Fractures	VDD	404	25	93.30%	0.980	(0.553, 1.739)	0.946

		VDA	343	22	93.35%			
3 years		VDD	404	57	82.92%	1.058	(0.721, 1.553)	0.773
		VDA	343	48	83.77%			
5 years		VDD	438	90	72.11%	1.115	(0.809, 1.536)	0.506
		VDA	338	64	74.45%			

Abbreviations: VDD, vitamin D deficiency; VDA, vitamin D adequacy; MACE, major adverse cardiovascular events; MAKE, major adverse kidney events; HR, hazard ratio; CI, confidence interval.

Supplementary Table S3. Severity of Vitamin D Deficiency and 5-Year Kaplan–Meier Outcomes in Patients with Sjögren's Syndrome and Osteoporosis. 5-year Kaplan–Meier survival analyses comparing patients with vitamin D deficiency (VDD; serum 25-hydroxyvitamin D <20 ng/mL) and vitamin D insufficiency (VDI; 20–29 ng/mL) versus those with vitamin D adequacy (VDA; ≥30 ng/mL).

Outcomes	Cohorts	Patients in cohort	Patients with outcome	Survival probability	Hazard Ratio	95% CI	Log-Rank test P-value
All-cause mortality	VDI	881	180	75.62%	1.938	(1.521, 2.468)	< 0.001
	VDA	905	103	86.26%			
	VDD	1,028	269	67.54%	3.400	(2.673, 4.326)	< 0.001
	VDA	1,053	88	87.83%			
MACE	VDI	513	181	58.80%	1.580	(1.263, 1.977)	< 0.001
	VDA	523	133	70.08%			
	VDD	473	157	56.86%	2.018	(1.589, 2.562)	< 0.001
	VDA	649	118	75.69%			

MAKE	VDI	801	202	70.24%	2.014	(1.601, 2.534)	< 0.001
	VDA	835	114	83.93%			
	VDD	862	250	63.50%	3.903	(3.045, 5.004)	< 0.001
	VDA	989	83	87.73%			
Fractures	VDI	397	85	73.10%	0.751	(0.558, 1.012)	0.059
	VDA	316	88	67.24%			
	VDD	438	90	72.11%	1.115	(0.809, 1.536)	0.506
	VDA	338	64	74.45%			

Abbreviations: VDD, vitamin D deficiency; VDI, vitamin D insufficiency; VDA, vitamin D adequacy; MACE, major adverse cardiovascular events; MAKE, major adverse kidney events; HR, hazard ratio; CI, confidence interval.

Supplementary Table S4. E-value Sensitivity Analysis for Primary Outcomes According to Vitamin D Status in Patients with Sjögren's Syndrome and Osteoporosis. E-values for the associations between vitamin D deficiency (VDD; serum 25-hydroxyvitamin D <20 ng/mL) and the primary outcomes, including all-cause mortality, major adverse cardiovascular events (MACE), major adverse kidney events (MAKE), and fractures, compared with vitamin D adequacy (VDA; ≥30 ng/mL).

Outcomes	Cohorts	Patients in cohort	Patients with outcome	Survival probability	Hazard Ratio	95% CI	Log-Rank test P-value
All-cause mortality	VDI	881	180	75.62%	1.938	(1.521, 2.468)	< 0.001
	VDA	905	103	86.26%			
	VDD	1,028	269	67.54%	3.400	(2.673, 4.326)	< 0.001
	VDA	1,053	88	87.83%			
MACE	VDI	513	181	58.80%	1.580	(1.263, 1.977)	< 0.001
	VDA	523	133	70.08%			
	VDD	473	157	56.86%	2.018	(1.589, 2.562)	< 0.001

	VDA	649	118	75.69%			
MAKE	VDI	801	202	70.24%	2.014	(1.601, 2.534)	< 0.001
	VDA	835	114	83.93%			
	VDD	862	250	63.50%	3.903	(3.045, 5.004)	< 0.001
	VDA	989	83	87.73%			
Fractures	VDI	397	85	73.10%	0.751	(0.558, 1.012)	0.059
	VDA	316	88	67.24%			
	VDD	438	90	72.11%	1.115	(0.809, 1.536)	0.506
	VDA	338	64	74.45%			

Abbreviations: VDD, vitamin D deficiency; VDA, vitamin D adequacy; MACE, major adverse cardiovascular events; MAKE, major adverse kidney events.

Supplementary Table S5. Longitudinal vitamin D exposure in patients with Sjögren's syndrome and osteoporosis. Serum 25-hydroxyvitamin D [25(OH)D] levels and the frequency of vitamin D deficiency diagnoses (ICD-10 code E55) were summarized during early (0–3 years) and late (4–5 years) follow-up after cohort entry.

Follow Up	Outcome	Cohorts	Patients in cohort	Patients with outcome	Mean serum 25(OH)D (ng/mL)	Standard Deviation	P-value
Early follow-up (0–3 years)	25(OH)D levelsz	VDD	770	231	11.444	3.818	<0.0001
		VDA	770	360	49.984	14.682	
Late follow-up (4–5 years)		VDD	654	39	10.916	4.006	<0.001
		VDA	654	118	54.023	19.758	
Early follow-up (0–3 years)	Number of VDD diagnosis	VDD	770	325	1.449	2.896	<0.0001
		VDA	770	206	0.879	2.293	

Late follow-up (4–5 years)		VDD	654	90	0.411	1.383	0.6128
		VDA	654	90	0.373	1.348	

Abbreviations: VDD, vitamin D deficiency; VDA, vitamin D adequacy.
